# Heterogeneous disease and intermittent treatment in metastatic colorectal cancer: A case report

**DOI:** 10.3389/fonc.2023.1084681

**Published:** 2023-02-06

**Authors:** Alfonso De Stefano, Nicoletta Zanaletti, Antonino Cassata, Lucrezia Silvestro, Anna Nappi, Rossana Casaretti, Carmela Romano, Francesca Foschini, Claudia Cardone, Marco Borrelli, Antonella Petrillo, Alfredo Budillon, Paolo Delrio, Antonio Avallone

**Affiliations:** ^1^ Experimental Clinical Abdominal Oncology Unit, Istituto Nazionale Tumori – IRCCS – Fondazione G. Pascale, Napoli, Italy; ^2^ Division of Radiology, Istituto Nazionale Tumori – IRCCS – Fondazione G. Pascale, Napoli, Italy; ^3^ Scientific Directorate, Istituto Nazionale Tumori – IRCCS – Fondazione G. Pascale, Napoli, Italy; ^4^ Colorectal Surgical Oncology, Istituto Nazionale Tumori – IRCCS – Fondazione G. Pascale, Napoli, Italy

**Keywords:** case report, anti-EGFR, resistance, intermittent strategy, RAS, BRAF, tumor heterogeneity, precision medicine

## Abstract

**Background:**

Metastatic colorectal cancer is one of the most common causes of cancer death worldwide. RAS and BRAF mutational analyses are strongly recommended before beginning chemotherapy in the metastatic setting for their predictive role for the efficacy of anti-EGFR monoclonal antibodies. In most of cases, mutational status coincides between primary tumor and metastases. In RAS and BRAF wild-type patients treated with anti-EGFRs, after an induction treatment period, recent evidence supports the role of a maintenance treatment with fluoropyrimidines and anti-EGFRs. However, skin toxicity is the most described and limiting side-effect of maintenance. Moreover, it is described that the continuous administration of these monoclonal antibodies leads to an acquired resistance to anti-EGFRs, with subsequent treatment failure. Intermittent strategy with chemotherapy plus anti-EGFR may help maintain treatment efficacy, delaying resistance.

**Case presentation:**

In this case report, we describe the case of a RAS-BRAF wild-type elderly patient undergoing first-line chemotherapy with FOLFOX + panitumumab, reporting response of disease on all metastatic sites except for a node. This node, surgically removed, revealed host BRAF V600 mutant clones. After surgery, patient continued chemotherapy with a stop-and-go strategy continuing to benefit from the same drugs after 4 years since diagnosis, and continuing to achieve response when on treatment, avoiding unacceptable anti-EGFR toxicity. This patient, still alive after 6 years since the diagnosis, represents the case of a good synergy between molecular profiling of disease, surgery, and intermittent treatment.

## Introduction

Colorectal cancer (CRC) is the third most common tumor in men and the second in women, accounting for 10% of all tumor types worldwide. With more than 600,000 deaths estimated each year, CRC is the fourth most commonly diagnosed cancer globally (1–3).

During the last years, the deeper knowledge of tumor biology and molecular genetics has considerably influenced the treatment and survival of metastatic colorectal cancer (mCRC) patients with the introduction of molecularly targeted agents. In particular, EGFR has emerged as a key target for CRC, and anti-EGFR monoclonal antibodies (cetuximab and panitumumab) combined with cytotoxic chemotherapy are standard treatments for RAS wild-type mCRC patients because of their clinical efficacy and prolonged survival (4–6).

RAS and BRAF analyses are considered mandatory before planning a treatment: several studies reported that RAS mutant mCRC patients are unlikely to benefit from anti-EGFR antibodies and BRAF V600 mutations were shown to predict the lack of clinically meaningful efficacy of EGFR inhibitors (7); therefore, such therapies are not recommended for RAS/BRAF mutants (8). Moreover, following the recent evidence of clinical trials and updated guidelines, deficient mismatch repair (dMMR)/microsatellite instability (MSI) testing is recommended to select patients for immune checkpoint inhibition (ICI) in the first-line setting (9). After progression from first-line, identification of HER2 amplification is recommended in RAS wild-type patients to detect those who may benefit from HER2 blockade (10), although anti-HER2 inhibition is only recommended in second and further lines (11).

Giving a deeper insight, even in RAS and BRAF wild-type patients, the emergence of resistant tumor cell populations occurs almost inevitably, leading to treatment failure (12). The development of resistance and the evolutionary ability of cancer to adapt to treatment perturbations stem from spatial and temporal molecular heterogeneity of the tumor (13).

In mCRC patients, clinical data highlighted that the tumor genome evolves dynamically during treatment, showing the emergence of resistant mutated RAS clones during EGFR blockade, and their decline after the interruption of the anti-EGFR pressure (14). Moreover, in non-small cell lung and colorectal cancers, preclinical data suggested that alternative to the intrinsic existence of resistant clones, *de novo* resistant clones can develop during the course of prolonged EGFR blockade (15, 16). In this scenario, to limit the development of resistance and achieve long-term effectiveness in mCRC, the optimized use of anti-EGFRs through adaptive therapeutic strategies, such as the intermittent application like the stop and go approach, could be adopted to delay the onset of resistance.

## Case presentation

**Figure 1:**
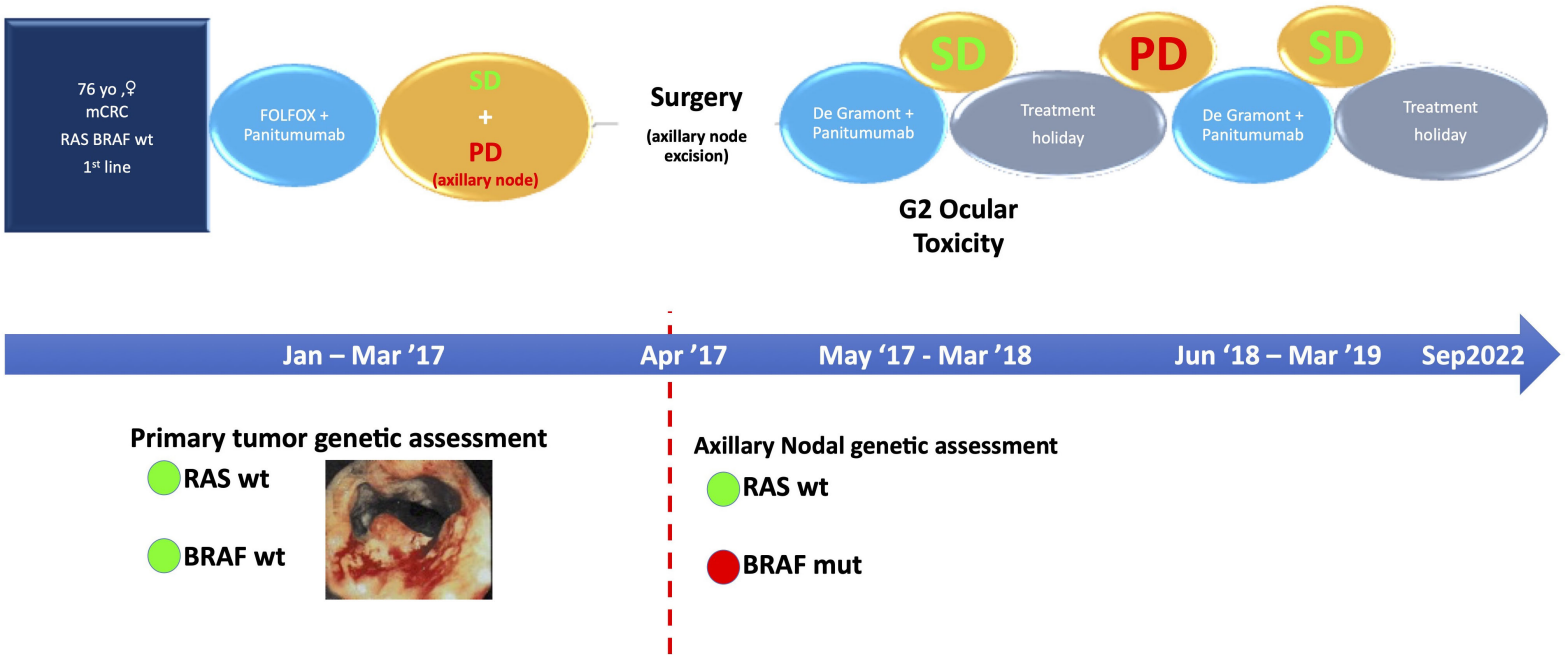
Specific time points corresponding to the diagnostic and therapeutic process. SD, stable disease; PD, progressive disease.

**Figure 2:**
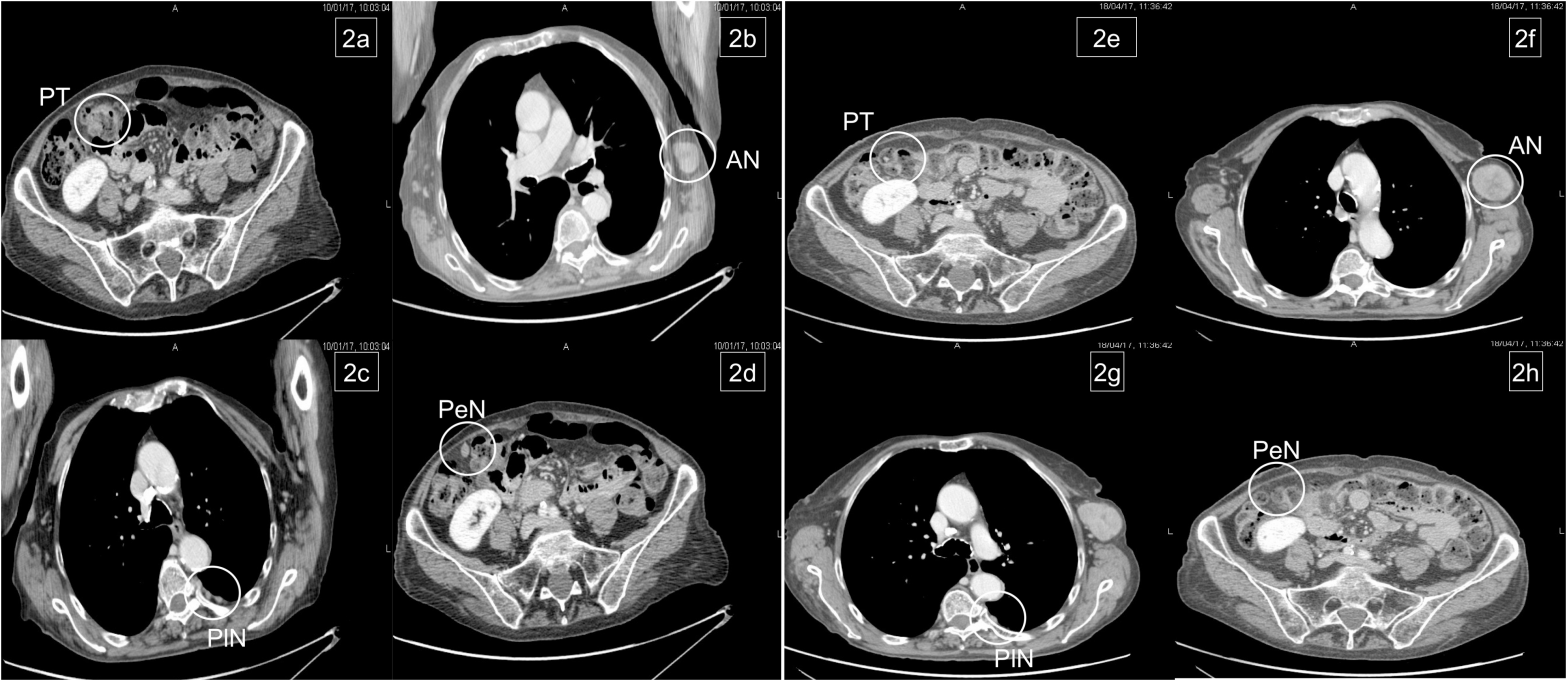
Basal metastatic sites at diagnosis: primary tumor (PT), left axillary node (AN), pleural nodes (PlN), and peritoneal node (PeN) **(A–D)**. Response to treatment after six courses: patient achieved response on primary tumor and all metastatic sites, except for the left axillary node **(E–H)**.

We report the case of a 76-year-old woman (specific time points in **Figure 1**) diagnosed in December 2016 with stage IV RAS BRAF wild-type, poorly differentiated transverse colon adenocarcinoma with subcutaneous, pleural, left axillary, and abdominal lymph-node involvement (**Figures 2A–D**). A colonoscopy sample was used to perform RAS BRAF analysis, adopting the Oncomine Solid Tumour panel on Next Generation Sequencing (NGS) Ion Torrent platform, with a limit of detection of 5%.

In January 2017, the patient began chemotherapy with mFOLFOX-6 plus panitumumab. After six cycles, she reported a mixed response for a partial remission on metastatic sites but progressive disease on the axillary node (**Figures 2E–H**). During these first 3 months, the patient reported G1 skin rash, G1 peripheral neuropathy, and G2 neutropenia as side-effects, according to CTCAE 4.0.

This case was evaluated by a multidisciplinary team. Since disease progression only occurred at a single site, surgical excision of the left axillary node was scheduled (April 2017). Histopathological findings were compatible with metastasis from intestinal adenocarcinoma and the molecular assessment detected a BRAF V600E mutation that was probably the cause of resistance to panitumumab.

After surgery, the patient restarted chemotherapy with mFOLFOX-6 plus panitumumab for another six cycles and then continued with 5-FU/FA (De Gramont schedule) plus panitumumab as maintenance until March 2018. Afterwards, for persistent conjunctivitis (Grade 2, CTCAE 4.0) and considering the disease control, confirmed by a quarterly CT-scan, the patient began a treatment holiday period.

**Figure 3:**
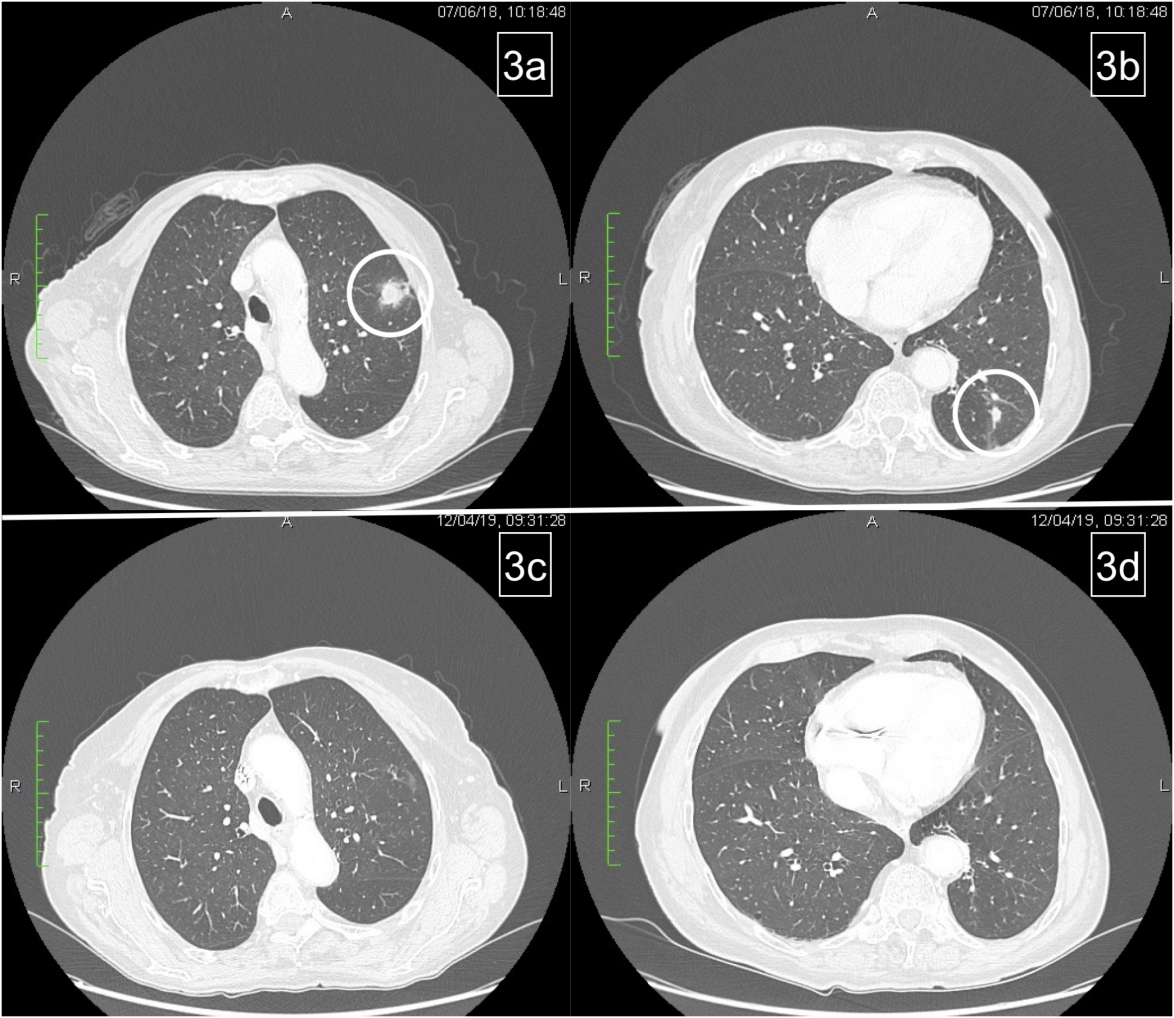
Pulmonary relapse **(A, B)**. Complete response of lung metastases after reintroduction of chemotherapy **(C, D)**.

On June 2018, CT scan showed lung progression for evidence of two new lesions (**Figures 3A, B**). Therefore, the patient resumed 5-FU/FA + panitumumab until March 2019. During this period, disease stability and response on the new lung metastases were achieved (**Figures 3C, D**) and treatment was stopped temporarily for ocular toxicity (Grade 2, CTCAE 4.0) that was particularly bothersome for the patient.

To date, with CT scan performed every two months, after 36 months since the last cycle and nearly 72 months since diagnosis, no evidence of disease progression has been observed and the patient’s clinical condition was also preserved.

## Discussion

Colorectal cancer is one of the most common malignancies in Western countries.

Molecular profiling of mCRC patients is mandatory before planning a treatment strategy as the initial choice will potentially influence survival. Mutational analysis of RAS and BRAF is on the basis of the molecular definition as the use of anti-EGFR antibodies in RAS-BRAF wild-type patients may help overcome a median survival time of 30 months.

Unfortunately, as formerly reported, tumor cells develop resistance towards anti-EGFRs, due to a selective pressure exerted by the continuous targeting of EGFR. One of the most stimulant challenges for oncologists is represented by understanding how to overcome this resistance or, at least, to mitigate and delay it.

In this report, an interesting aspect is represented by the spatial heterogeneity for the incomplete concordance between metastatic sites and primary tumor. KRAS mutations are generally believed to be early events in CRC carcinogenesis and thus high concordance between primary tumor and metastatic lesions is expected (17). A systematic review established a high concordance rate of KRAS mutational status between primary tumor and metastases (93%) (18).

As reported here, mutational status can be discordant among the primary tumor and nodes. As described in literature, nodal metastases in colon cancer are polyclonal (19–21).

These reports underline the need to consider spatial heterogeneity while treating a disease and as we report, recurring surgery is a strategy to overcome it. In fact, by removing the only metastatic site discordant with the mutational status of the tumor, the efficacy of panitumumab was preserved.

In mCRC patients receiving anti-EGFRs, the tumor genome evolves, dynamically promoting the spread of initially silent mutant subclones.

The selective pressure exerted by anti-EGFRs is considered one of the most consistent causes of resistance, as it could stimulate the increase of initially silent resistant clones that, however, decay in a time-dependent trend after discontinuation of anti-EGFR (22).

On the basis of such hypotheses, intermittent use of anti-EGFRs may represent a valid option to preserve efficacy and to prolong clinical benefit. Moreover, intermittent treatment may avoid the increase of typical toxicity such as skin rash and paronychia, encountering a better compliance of patients towards treatment. These were the reasons and rationale for which we adopted the intermittent therapeutic strategy for the treatment of our patient.

In this regard, a meta-analysis showed that intermittent strategies of administering first-line treatment to patients with unresectable mCRC do not result in a statistically significant reduction in overall survival (OS), and either improve or maintain quality of life (23). Moreover, the feasibility of intermittent use of chemotherapy was demonstrated in some clinical trials. The GISCAD study, which randomized 337 patients to receive intermittent or continuous treatment with FOLFIRI until disease progression, showed that the intermittent schedule was not inferior to continuous treatment for progression-free survival and OS (24).

While maintenance therapy with anti-VEGF agents is well coded until disease progression, no data are available on the optimal duration of anti-EGFRs. The VALENTINO trial (25) showed the superiority of the maintenance with panitumumab plus 5FU/FA compared with panitumumab alone after an induction treatment with FOLFOX plus panitumumab. Recently, an interesting *post hoc* analysis of this trial was performed on patients experiencing progressive disease not on treatment, undergoing a conventional second-line chemotherapy, or reinduction with anti-EGFR (all patients had an anti-EGFR free interval of at least 3 months). Patients receiving reinduction obtained a similar progression-free survival (PFS) but achieved a significant longer OS and higher response rate (RR) (26).

The recent results of PANAMA trial (27), where patients were randomized to maintenance treatment with 5FU/FA with or without panitumumab, showed an improved PFS and RR for subjects continuing anti-EGFR after induction chemotherapy. According to the design of this trial, patients progressing during maintenance treatment, received reinduction chemotherapy with FOLFOX plus panitumumab. PFS of reinduction therapy with FOLFOX plus panitumumab was 3.8 months (95% CI, 2.5–4.8) versus 6.3 months (95% CI, 4.7–8.2) in patients who had received FU/FA and panitumumab versus FU/FA alone as maintenance therapy (HR, 2.34; 95% CI, 1.54–3.56; p = 0.001). The temporary discontinuation of panitumumab during maintenance in the control arm of the PANAMA trial translated into an advantage in PFS when it was restarted.

As a contribution, our group recently presented the positive results of the IMPROVE study, a prospective, randomized, non-comparative, open-label, multicenter phase II study (28), with PFS on treatment (PFS_OT_) as primary end-point. Unresectable, previously untreated RAS/BRAF wild-type mCRC patients were randomized to receive FOLFIRI plus panitumumab continuously until progression or eight cycles of the same regimen followed by a treatment free interval. This lasted until progressive disease, when another treatment period of eight cycles was restarted. This intermittent strategy was continued until progression occurred on treatment. Final results showed that the median PFS_OT_ was 12.6 months in the continuous arm and 17.6 months in the intermittent arm, with 1-year PFS_OT_ rates of 51.7% and 61.3%, respectively.

In conclusion, we would underline the importance of multidisciplinary choices as the acquisition of new intrinsic aspects, such as spatial and temporal heterogeneity, can translate into more effective treatment strategies.

In managing the case of our patient, as there was only one site of progression, excisional surgery helped us eradicate the only metastatic site not responding to the anti-EGFR antibody as the BRAF mutant and let us continue the same treatment with a great advantage for the patient, thanks to the intermittent strategy.

## Data availability statement

The raw data supporting the conclusions of this article will be made available by the authors, without undue reservation.

## Ethics statement

Written informed consent was obtained from the individual(s) for the publication of any potentially identifiable images or data included in this article.

## Author contributions

AA and AD provided ideas for this case. AD, AN, NZ, and AA drafted the manuscript. AP provided figures. AD, AN, MB, AB and AA acquired, analyzed, and interpreted the data. AC, LS, RC, CR, FF, CC, and PD diagnosed the disease and followed the patient during treatment and follow up. All authors revised the manuscript critically for important intellectual content and agreed to be accountable for all aspects of the work in ensuring that questions related to the accuracy or integrity of any part of the work are appropriately investigated and resolved. All authors contributed to the article and approved the submitted version manuscript.
